# Impact of probiotics use on clinical outcomes of immune checkpoint inhibitors therapy in cancer patients

**DOI:** 10.1002/cam4.4994

**Published:** 2022-06-30

**Authors:** Luying Wan, Chunlan Wu, Qin Wu, Shuimei Luo, Junjin Liu, Xianhe Xie

**Affiliations:** ^1^ Department of Oncology The First Affiliated Hospital of Fujian Medical University Fuzhou Fujian China; ^2^ Department of Infectious Diseases The First Affiliated Hospital of Fujian Medical University Fuzhou Fujian China; ^3^ Fujian Key Laboratory of Precision Medicine for Cancer The First Affiliated Hospital of Fujian Medical University Fuzhou Fujian China

**Keywords:** gut microbiome, immune checkpoint inhibitors, objective response rate, overall survival, progression‐free survival, probiotics

## Abstract

**Background:**

Dysbiosis of the gut microbiota can lead to impaired therapeutic effect of immune checkpoint inhibitors (ICIs). This study aimed to investigate the use of probiotics on the clinical outcomes of cancer patients receiving ICIs therapy.

**Method:**

PubMed, EMBASE, and the Cochrane Library database were searched to retrieve relevant studies that exploring the relationship between probiotics and the efficacy of ICIs. The primary endpoints included overall survival (OS) and progression‐free survival (PFS), evaluated by the hazard rations (HRs) with 95% confidence intervals (CI), and the secondary endpoint was objective response rate (ORR), evaluated by the odd ratio (OR) with a 95% CI.

**Results:**

A total of five studies including 1031 patients were eligible for analysis. Our results indicated that the use of probiotics was associated with a superior OS (HR = 0.50, 95% CI: 0.30–0.85, *p* = 0.01) and PFS (HR = 0.51, 95% CI: 0.42–0.61, *p* < 0.01), but had no relationship with ORR (OR = 2.11, 95%CI: 0.51–8.65, *p* = 0.30) in non‐small cell lung cancer (NSCLC) patients.

**Conclusions:**

Probiotics were positively correlated with OS and PFS in NSCLC patients administrated with ICIs, but had no relationship with ORR.

## Graphical abstract

Considering that the composition of the gut microbiota can dictate the clinical benefits of immune checkpoint inhibitors (ICIs), this study examined whether the use of probiotics can affect clinical outcomes of cancer patients receiving ICIs therapy. The results indicated that probiotics were positively correlated with overall survival (OS) and progression‐free survival (PFS) in non‐small cell lung cancer (NSCLC) patients administrated with ICIs, but had no relationship with objective response rate (ORR).

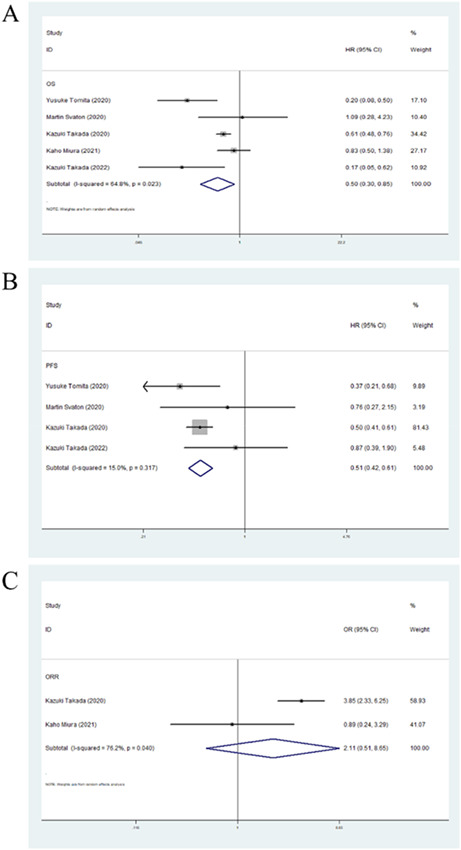

## 1 INTRODUCTION

Immune checkpoint inhibitors (ICIs) have revolutionized the treatment paradigm of various malignancies and become their standard of care.^1^, ^2^, ^3^ Still, only a proportion of patients exhibit response to ICIs and the drug efficacy presentations may vary among individuals. Existing biomarkers, such as programmed cell death‐ligand 1 (PD‐L1), mismatch repair (MMR) deficiency, and tumor mutational burden (TMB), were reported to have limited precision to predict clinical outcomes of patients treated with ICIs.^4^, ^5^ Therefore, it is particularly crucial to accurately identify other determinants of the therapeutic efficacy of ICIs.

Recently, reports have depicted evidence that the composition of the gut microbiota can dictate the clinical benefits of ICIs.^6^ Matson et al showed that, in melanoma patients who were administrated with PD‐1 antibody, responders had more abundant fecal bacterial species than non‐responders, and germ‐free mice transplanted with fecal bacteria from responding patients showed activated immune response and improved efficacy of anti‐PD‐L1 therapy.^7^ In addition, our previous meta‐analysis indicated that dysbiosis of the gut microbiota by use of antibiotics can lead to resistance to ICIs and diminished clinical efficacy in a variety of cancers.^8^ These findings suggested the possibility of manipulating commensal microbiota to enhance ICIs efficacy, indicating that adding probiotics to the treatment regime might be a promising strategy to improve the clinical outcomes of ICIs. However, there is no yet a consensus focusing on the correlation between probiotics and ICIs, which needs further investigation.

Probiotics, as components of intestinal flora, are bacteria that can confer health benefits on the host when given in adequate amounts, and are reported to be involved in various physiological and pathological processes including alleviating inflammation and improving immune function.^9^, ^10^, ^11^, ^12^ However, recent researches yielded controversial results regarding the role of concomitant use of probiotics on the therapeutic effect of ICIs. Therefore, a meta‐analysis is required. To our knowledge, this is the first meta‐analysis addressing this problem, which might help clinicians to drive therapeutic decision‐making on whether to combine probiotic therapy with ICIs.

## 2 MATERIALS AND METHODS

### 2.1 Search strategy

This study was designed and performed in accordance with the Preferred Reporting Items for Systematic Reviews and Meta‐Analysis (PRISMA) guidelines. Articles were identified through electronic search in three databases (EMBASE, Pubmed/Medline, and the Cochrane Library), using the cut‐off date of February 10, 2022, with no limitation of languages. After that, the titles and abstracts of articles were screened preliminarily. The search terms utilized were as follows: “immune checkpoint inhibitors”, “immune checkpoint blockade”, “immune checkpoint blocker”, “immune checkpoint inhibitor”, “PD‐L1 inhibitor”, “PD 1 inhibitor”, “programmed cell death protein 1 inhibitor”, “programmed death ligand 1 inhibitor”, “programmed death ligand 1 inhibitor”, “CTLA 4 inhibitor”, “CTLA‐4 inhibitor”, “cytotoxic t lymphocyte associated protein 4 inhibitor”, “cytotoxic T lymphocyte associated protein 4 inhibitor”, “PD‐1 blockade”, “PD‐L1 blockade”, “nivolumab”, “ipilimumab”, “tremelimumab”, “pembrolizumab”, “atezolizumab”, “avelumab”, “durvalumab”, “bintrafusp alfa”, “toripalimab”, “envafolimab”, “sintilimab”, “cemiplimab”, “tislelizumab”, “camrelizumab”, “cetrelimab”, “pidilizumab”, “probiotic”, “lactobacillus”, “bifidobacterium”, “concomitant medication”.

### 2.2 Study selection

Studies that met all of the following criteria were included: (1) Patients: Patients were pathologically diagnosed as solid malignant tumors, and had received ICI therapy alone or in combination; (2) intervention: Probiotics were administered before, during, or after the ICIs therapeutic course in the experimental group, but no probiotic were prescribed in the control group; (3) outcome: A hazard ration (HR) with 95% confidence interval (CI) for overall survival (OS) and/or progression‐free survival (PFS) and/or odds ratios (OR) with 95% CI for objective response rate (ORR) could be extracted or calculated from the literature.

### 2.3 Data extraction and quality evaluation

The Newcastle‐Ottawa Quality Assessment Scale (NOS) was adopted to assess the quality of each study and studies with five or more stars were regarded as medium to high quality and were included finally. Two investigators independently screened and evaluated the studies according to inclusion criteria and extracted data from the recruited studies. Any discrepancies were resolved after discussion among all authors. Information was collated from the eligible studies: The first author's name, year of publication, type of cancer, country, sample size, type of ICIs and probiotics, study design, probiotics use window, OS with 95% CI, PFS with 95% CI, and ORR with 95% CI.

### 2.4 Statistical analysis

The primary outcomes of this study were OS and/or PFS and the secondary endpoint was ORR. The pooled HRs/ORs and 95% CI were calculated to indicate the effect of probiotics administration on ICIs efficacy. Statistical analysis was conducted with Stata version 12. The *I* ^2^ tests was performed to evaluated the heterogeneity among the recruited studies. Substantial heterogeneity existed when *I* ^2^ > 50% or *p* < 0.1, in which case a random‐effects model was used, otherwise a fixed‐effects model was applied. *p* < 0.05 was set as statistically significant.

## 3 RESULT

### 3.1 Search results and study characteristics

**FIGURE 1:**
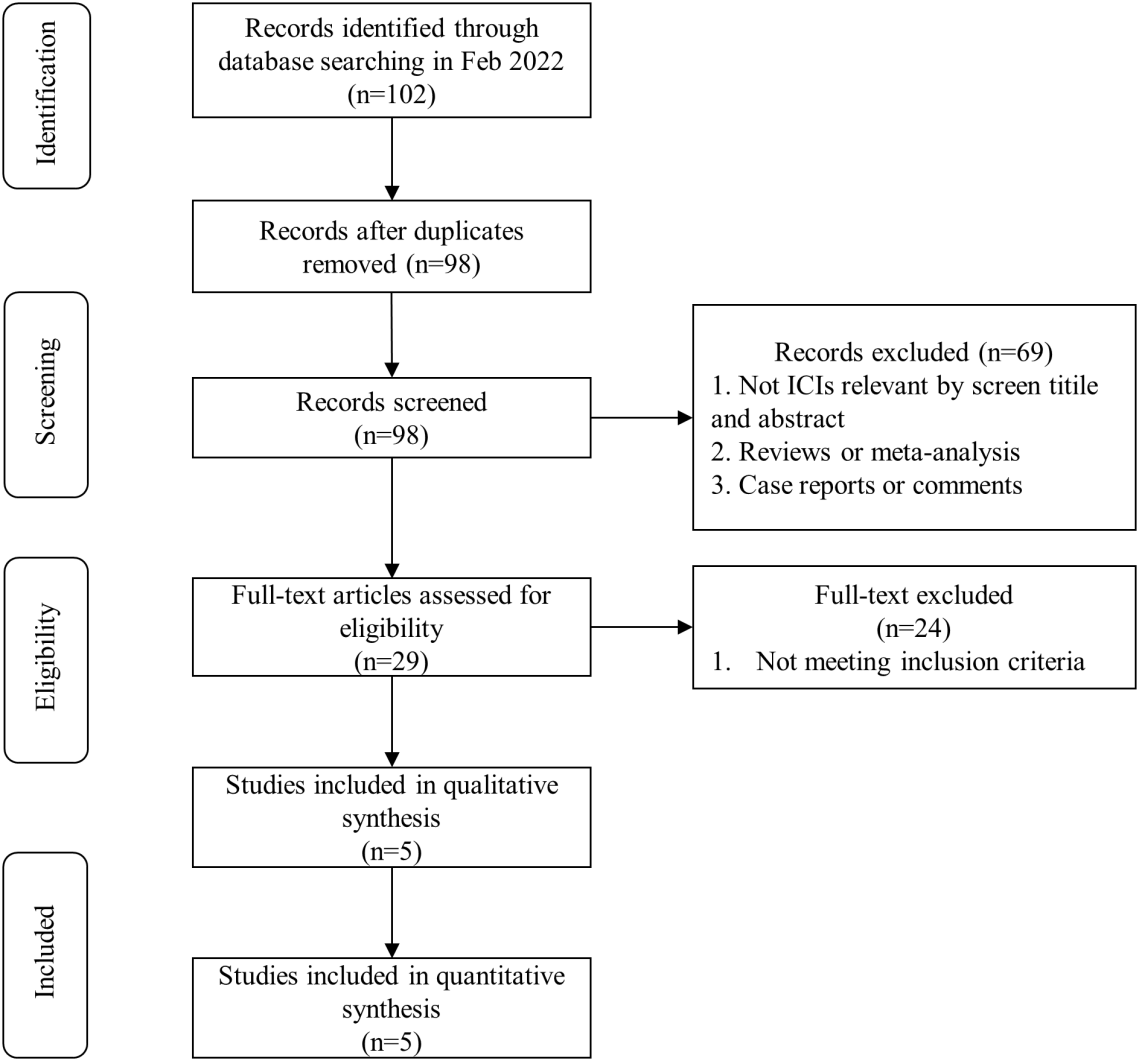
Flow diagram of study inclusion procedure.

A total of 102 relevant articles were initially acquired from the database, and four duplicates were removed. By meticulously scanning titles and abstracts, 69 were excluded due to not ICIs relevant studies, reviews, meta‐analyses, case reports, and comments, and 29 articles were obtained for further study. Of those, 24 articles did not meet the inclusion criteria. Finally, five studies were identified as eligible for inclusion (Figure 1).

All the included studies were retrospective and were published between 2020 and 2022. Among the five studies, four studies were conducted in Japan^13^, ^14^, ^15^ and one in Czech Republic.^16^ A total of 1031 patients with advanced or recurrent non‐small cell lung cancer (NSCLC) were enrolled in the five studies, including 118 patients in study Yusuke Tomita (2020), 224 in Martin Svaton (2020), 294 in Kazuki Takada (2020), 300 in Kaho Miura (2021), 95 in Kazuki Takada (2022). Meanwhile, all the patients received anti‐PD‐1 or anti‐PD‐L1: Nivolumab in all the five studies, Pembrolizumab in four, or Atezolizumab in two. Two studies described reasons for the use of probiotics, including diarrhea, loose stool, constipation, non‐specific abdominal symptoms, antibiotics‐associated dysbiosis, immune‐related enterocolitis, and unknown reason. *Lactobacillus* was used in all the five studies, Butyric Acid Bacteria (or *Clostridium butyricum*) in four, *Bifidobacterium* in three. Probiotic use window ranged from 6 months before ICIs treatment to after treatment, but the information of one study was unavailable. Five studies were provided with the effect of probiotic use on OS, four on PFS, and two on ORR. The baseline characteristics of these enrolled studies are summarized in Table 1.

**TABLE 1:** The characteristics of recruited studies in this meta‐anaysis

Author	Year	Country	Cancer type	Study type	Sample(Y/N)	Type of ICIs	Type of probiotics	Indication of probiotics	Probiotic use window	Outcomes HR (95%CI)		Outcomes OR (95% CI)
										OS	PFS	ORR
Yusuke Tomita	2020	Japan	NSCLC	Retro	118 (39/79)	Nivo, Pembro, Atezo	*Clostridium butyricum*	NA	Within 6 months before or concurrent with ICI treatment	0.20 (0.08–0.50)	0.37 (0.21–0.68)	NA
Martin Svaton	2020	Czech Republic	NSCLC	Retro	224 (6/218)	Nivo	Lactobacillus	NA	Within 1 month before or after ICI treatment	1.09 (0.28–4.23)	0.77 (0.27–2.15)	NA
Kazuki Takada	2020	Japan	NSCLC	Retro	294 (32/262)	Nivo, Pembro	Bifidobacterium, *Clostridium butyricum*, Lactic acid bacteria	Diarrhea, loose stool, constipation, unknown,	At the time of ICI treatment initiation	0.61 (0.48–0.76)	0.50 (0.41–0.61)	3.85 (2.33–6.25)
Kaho Miura	2021	Japan	NSCLC	Retro	300 (14/286)	Nivo, Pembro	Antibiotics‐resistant lactic acid bacteria, Bifidobacterium, butyric acid bacteria	NA	NA	0.83 (0.50–1.38)	NA	0.89 (0.24–3.29)
Kazuki Takada	2022	Japan	NSCLC	Retro	95(12/83)	Nivo, Pmbro, Atezo	Bifidobacterium, *Clostridium butyricum*, lactic acid bacteria	Diarrhea, Constipation, Non‐specific abdominal symptoms, Antibiotics‐associated dysbiosis, Immune‐related enterocolitis	At the time of ICI treatment initiation	0.17 (0.05–0.62)	0.87 (0.39–1.91)	NA

Abbreviations: Atezo, Atezolizumab; ICIs, immue checkpoint inhibitors; NA, not available; Nivo, Nivolumab; NSCLC, non‐small cell lung cancer; OS, overall survival; Pembro, Pembrolizumab; PFS, progression free survival; Retro, Retrospective; Y/N, probiotic use/ no probiotic use.

### 3.2 Quality assessment

According to the NOS criteria, all the recruited articles were rated by two reviewers as moderate or high quality, with the score ranging from five to nine. Therefore, all of the five studies were regarded as eligible for meta‐analysis. The NOS score of the included studies in this article are shown in Table 2.

**TABLE 2:** Quality assessment of included studies

Studies	Representativeness of population	Non‐respondents	Ascertainment of the exposure	Outcome of interest was not present at start of study	Comparability of cohorts on the basis of the design or analysis	Assessment of outcome	Enough follow‐up period	Adequacy of follow‐up of cohorts	Total stars
Yusuke Tomita (2020)	☆	☆	☆	☆	☆☆	☆	☆	☆	9
Martin Svaton (2020)	—	☆	☆	☆	☆	☆	☆	—	6
Kazuki Takada (2020)	☆	☆	☆	☆	☆	☆	☆	—	7
Kaho Miura (2021)	—	☆	☆	☆	☆	‐	☆	—	5
Kazuki Takada (2022)	☆	☆	☆	☆	‐	☆	☆	—	6

☆, The score of the item; —, no score in this item.

### 3.3 Main results

**FIGURE 2:**
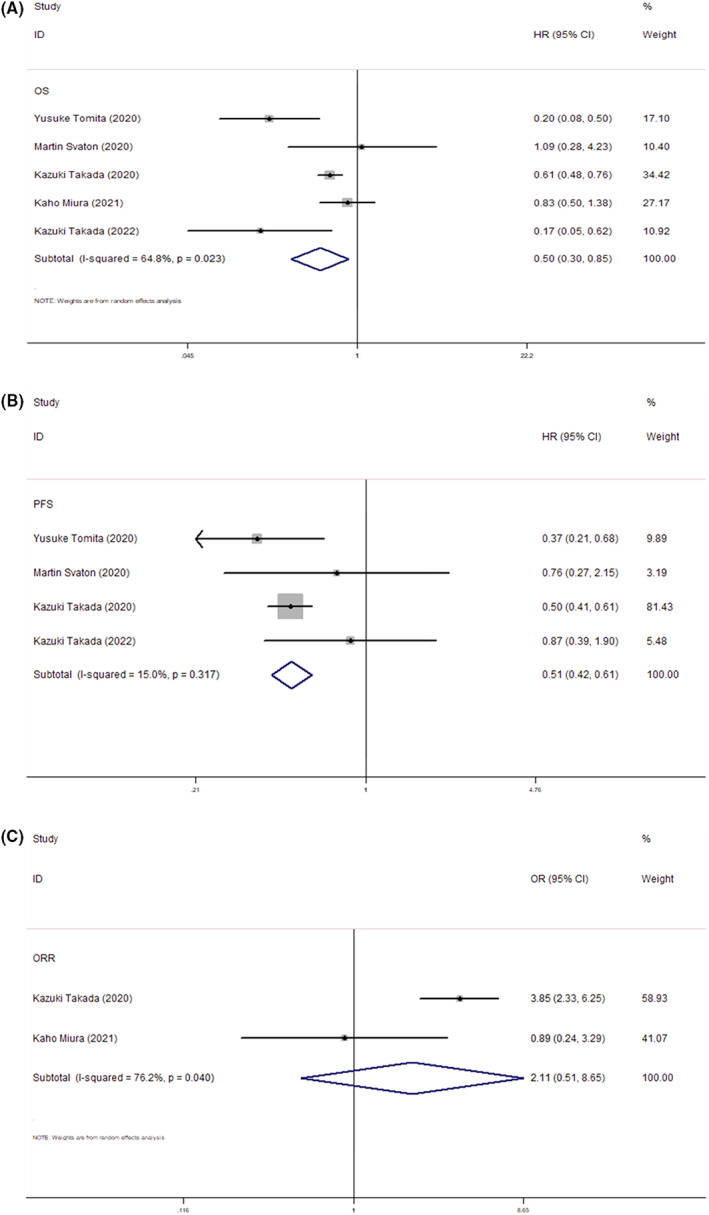
Forest plots for (A) overall survival, (B) progression‐free survival, and (C) objective response rate.

Probiotics use was significantly associated with a better OS in NSCLC patients receiving anti‐PD‐L1 or anti‐PD‐1 therapy (HR = 0.50, 95% CI: 0.30–0.85, *p* = 0.01). A random‐effect model was applied because of high heterogeneity (*I* ^2^ = 64.8%, *p* = 0.023). The administration of probiotics was also correlated with a better PFS (HR = 0.51, 95% CI: 0.42–0.61, *p* < 0.01) in our study, by a fixed‐effect model (*I* ^2^ = 15.0%, *p* = 0.317). However, probiotic had no relationship with ORR (OR = 2.11, 95% CI: 0.51–8.65, *p* = 0.30) by a random‐effect model (*I* ^2^ = 76.2%, *p* = 0.04) (Figure 2).

Subgroup analyses of the impact of type of probiotics, ICIs, or the use of antibiotics were not possible due to limited recruited studies and unavailability of data.

Publication bias analysis revealed no evidence of publication bias by Egger's test in the analysis of OS and PFS, indicating our results were reliable (*p* < 0.05). The publication bias was not examined in the analysis of ORR because of an insufficient number of studies (2 articles).

**FIGURE 3:**
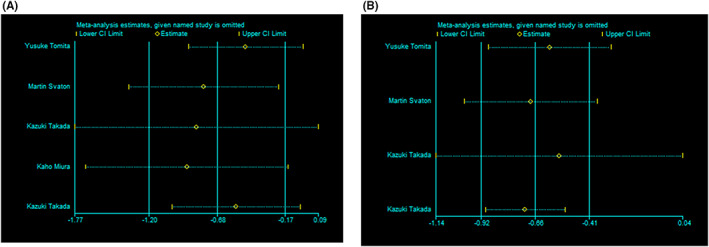
Sensitivity analysis of (A) overall survival and (B) progression‐free survival.

The sensitivity analyses were conducted by excluding one single study from the primary analyses, and the results revealed that no single study significantly influenced the pooled HRs, indicating that the data of this meta‐analysis were relatively credible and stable (Figure 3).

## 4 DISCUSSION

Previous studies have demonstrated that the gut microbiota is indispensable in the regulation of host immune response and is closely related to the efficacy of ICIs.^17^, ^18^ Researchers investigated the intestinal flora of patients receiving anti‐PD‐1/PD‐L1 therapy and found that the diversity or abundance of gut microbiota in responders was remarkably higher than that in non‐responders.^6^, ^19^ Besides, through fecal microbiota transplantation (FMT) transferring processed fecal material from melanoma patients who were ICIs sensitive to germ‐free mice, investigators found the effectiveness of PD‐1 inhibitor therapy in mice was obviously enhanced.^20^ Therefore, it is tempting to propose that probiotics, which have the potential to influence the gut microbiota, might improve ICIs efficacy. In preclinical studies, providing tumor‐bearing murine models with *Bifidobacterium pseudolongum, Lactobacillus johnsonii*, and *Olsenella* ^19^, ^21^, ^22^ could activate antitumor immunity and thereby increase ICIs therapeutic effect. Furthermore, in a Phase 1 clinical trial, FMT is able to re‐establish efficacy of ICIs in patients with metastatic melanoma refractory to these agents,^23^ supporting the idea of increasing efficacy of immunotherapy by modulating the intestinal flora. However, the role of probiotics supplementation in humans remains obscure, and this meta‐analysis was performed to assess this problem.

Probiotics, represented by *Bifidobacterium* and *Lactobacillus*, are key components of the normal intestinal flora that can help to prevent the development and progression of tumors.^24^, ^25^In recent years, the role of probiotics in tuning of immune response are being actively investigated.^26^, ^27^ The possible mechanisms of probiotics contributing to the improved response to ICIs are as follows: Firstly, probiotics possesses the ability of activating phagocytes to remove early‐stage cancer cells and regulating the production of anti‐inflammatory cytokines implicated in prevention of carcinogenesis,^28^ indicating an underlying promoting effect of ICI therapy. Secondly, the capability of probiotics affecting the efficacy of ICIs is closely related to the bacteria's regulation of the host's immunological background. For example, *Bifidobacterium bifidum* could enhance CD8+ T cells accumulation by augmenting dendritic cell function^21^ and stimulate the production of interferon‐γ,^19^ probably through increased biosynthesis of immune‐stimulating molecules and metabolites, facilitating the anti‐PD‐1/PD‐L1 efficacy. Bertrand Routy et al showed that oral supplement of *Akkermansia muciniphila* improved the efficacy of anti‐PD‐1 therapy through the interleukin‐12 (IL‐12)‐dependent recruitment of CCR9+CXCR3+CD4+T cells.^6^ The team also verified that the composition of *Bacteroides fragilis* and/or *B*acteroides *thetaiotaomicron* and *Burkholderiales* influenced interleukin 12 (IL‐12)–dependent T helper (TH_1_) immune responses, which contributed to tumor control of CTLA‐4 blockade in mice and patients.^29^ Finally, some strains of probiotics like *Lactobacillus* and *Bifidobacterium* could produce lactic and acetic acids, which lower luminal PH and induce resistance to colonization of genotoxic microbes in an ICI‐independent manner.^30^

The present study displayed that probiotics were significantly associated with a superior OS and PFS in NSCLC patients receiving ICIs, suggesting the potential benefits of adding probiotics into ICI therapy regime. However, our results failed to show statistical significance between probiotics with ORR. This may be mainly ascribed to the limited studies (only two) reporting the results of ORR and their small total sample size, and it is possible that conclusion may have differed if more studies enrolled in the future.

Previous researches have revealed that the use of antibiotics can disrupt the balance of intestinal microflora and impair the therapeutic effect of ICIs. Two of the five included studies discussed the influence of antibiotics on the relationship of probiotic and the clinical outcomes of ICIs. On the one hand, in cancer patients without antibiotic therapy, study Yusuke Tomita (2020) found that probiotics were associated with improved OS but not PFS, while Kazuki Takada (2020) came to an opposite result that probiotics were correlated with improved PFS but not OS. On the other hand, in patients treated with antibiotics, both studies showed positive association between probiotics and improved PFS and OS. These researches have provided a clue that probiotics may increase the efficacy of ICIs treatment for cancer patients who received antibiotics by regulating the gut microbiota disturbance caused by antibiotic therapy. However, only one of the two studies provided the HR values and 95% CI, which was insufficient to calculate the pooled HRs with 95% CI in the present meta‐analysis. Therefore, further correlative studies of the gut microbiota and randomized prospective trials will be needed to verify the hypothesis.

This study is subject to several limitations: First, the current meta‐analysis consisted of only retrospective studies but not randomized controlled trial (RCT). Second, since this article is the first meta‐analysis, as far as we know, to evaluate the impact of probiotics on clinical outcomes of ICIs in patients with cancer, it is expected to find a small number of studies. Although 1031 cases were included in our research through a comprehensive electronic literature search, only five studies were eligible for the final analysis, thus the subgroup analyses (such as geographic region, ICIs type, or probiotics type) was unable to perform. On the other hand, limited studies in meta‐analyses compromise the precision of the results that measure heterogeneity, which may lead to higher *I* ^2^ values. Therefore, our findings regarding the relationship between probiotics and ICIs are preliminary, which should be interpreted with caution, and further prospective clinical trials are needed.

In conclusion, our present study shows that the probiotics use is positively associated with a better OS and PFS in NSCLC patients undergoing ICI therapy. This result provides preliminary evidence to support combination of probiotic with ICIs treatment in these patients. Future researches are essential to examine this relationship in larger populations with different clinical characteristics.

## AUTHOR CONTRIBUTIONS

Luying Wan, Chunlan Wu, and Xianhe Xie: Study design; Luying Wan, Chunlan Wu, Qin Wu, Shuimei Luo, and Junjin Liu: data collection and analysis; LYW: writing of the first draft of the manuscript; Chunlan Wu, Xianhe Xie: revision of the draft; All authors involved in the final approval of the draft.

## CONFLICT OF INTEREST

The authors declare no conflict of interest.

## Data Availability

Data sharing is not applicable to this article as no new data were created or analyzed in this study.
